# Determinants of severe acute malnutrition among children aged 6–36 months in Kalafo district (riverine context) of Ethiopia

**DOI:** 10.1038/s41598-022-09184-y

**Published:** 2022-03-25

**Authors:** Ahmed Tahir Ahmed, Abdulahi Haji Abas, Abdifatah Elmi, Abdilahi Omer

**Affiliations:** Public Health Department, College of Medicine and Health Science, Jigjiga University, P.O. Box 1020, Jijiga, Ethiopia

**Keywords:** Health care, Medical research, Risk factors

## Abstract

Malnutrition remains prevalent and existing health problem globally. Particularly Undernutrition is a major public health issue in developing countries. Globally the causes of severe acute undernutrition varies across context. The aim of this study was to identify the determinants of severe acute malnutrition to uncover contextual factors based on UNICEF conceptual framework, as there was no study done in a similar context in Ethiopia. Health facility based (health post) un-matched case control study with Key informant interview was conducted to identify determinant factors of severe acute malnutrition (SAM) among children between 6 and 36 months. 246 children (82 cases and 164) with their mothers or care takers participated the study which was conducted between December 20, 2019 to January 20, 2020 in Kalafo district in Shebele River. Odds ratio with 95% confidence interval was calculated to identify the determinants of SAM among children aged 6–36 months using multivariate logistic regression. The odds of severe acute malnutrition was 2.28 (1.22, 4.26); 4.68 (2.29, 9.58); 2.85 (1.26, 6.45); 2.39 (1.16, 4.96) and 3.262 (1.46, 7.31) and 3.237 (1.45, 7.23); respectively for mothers with three or more under five children, Children with inadequate dietary diversity, experienced diarrhea in past 2 weeks, their mothers had not nutrition counselling during pregnancy and younger (6–11 and 12–17 months) children as compared to controls. The finding of this study reveals the main determinants of severe acute malnutrition in riverine context are multi-level. In addition to this, poor childcare and polygamy identified in qualitative finding. Decisive and multi-sectoral approach is required to addressing SAM in the riverine area.

## Introduction

Malnutrition remains prevalent and existing health problem globally^1–3^. Particularly Undernutrition is a major public health issue in low and middle income countries^2,4–6^. Across regions there are huge disparities regarding undernutrition. South Asia (35.8% and east Africa (34.4%) shows exceedingly higher stunting rate than global average (22.9%)^2^.

The trends of acute malnutrition (Wasting) remains the same over decades in Kenya and Zambia^7^. Despite national nutrition commitments, undernutrition among under-five children is a formidable challenge in Ethiopia too^8–11^, particularly in Somali region where wasting (22.7%) and anemia (83%) among under five children is profoundly higher than national average^8–10^. Recently, Somali region shared the highest (26%) severe acute malnutrition burden in the country^12^.

Globally the causes of acute undernutrition are too many and varies across context. Exhaustive identification of such broad multi-sectoral factors will lead comprehensive intervention. Many studies in developing countries including Ethiopia showed that household characteristics like income^13–15^, less access to health service^14^ household head^10,13,14^ lack of toilet^16^, mothers education^7,17^, paternal education^18^ handwashing^17,19^, household food insecurity^20^, poor exclusive breast feeding^21,22^, and number of under-five children or family size^17,23^ has explained the occurrence of acute malnutrition.

Moreover, many more factors like post-natal vitamin A supplementation^24^, acute infections like diarrhea and malaria^17,25–27^, child spacing^10^, maternal knowledge and awareness about infant and young child feeding^14^ iodized salt use^26^, water sources^10^, low birth weight^21^, birth order^28^, bottle feeding^29^, inappropriate initiation time of complementary feeding^15,20^, age of the child^27,30^, and low (< 45 kg) maternal weight^21^ are also behind the occurrence of acute malnutrition. The other important determinant of acute malnutrition were sex^31,32^ and inadequate dietary diversity of the child^21^. Likewise, recent studies indicate that feeding practice, maternal age and nutrition counseling during pregnancy were significantly associated with acute malnutrition among under-five children^29^^,^^33^. However, un-intended pregnancy increases the odds of chronic undernutrition (stunting) among under-five children^34^. Whereas; fathers and mothers literacy, and higher wealth index decrease the odds of under-nutrition among under-five children^11,34^.

The trends of severe acute malnutrition in Kalafo district of Shebele zone remains high according to the routine therapeutic feeding program reports (TFP). The number of TFP admissions in Kalafo district was two times higher compared to Mustahil district—an adjacent woreda with the same livelihood zone (unpublished save the children report, 2019). Moreover, unpublished SMART survey conducted in Kalafo district, April 2018 shows high (16%) GAM rate. This shows addressing only food insecurity will not guarantee addressing malnutrition in this area.

**Figure 1:**
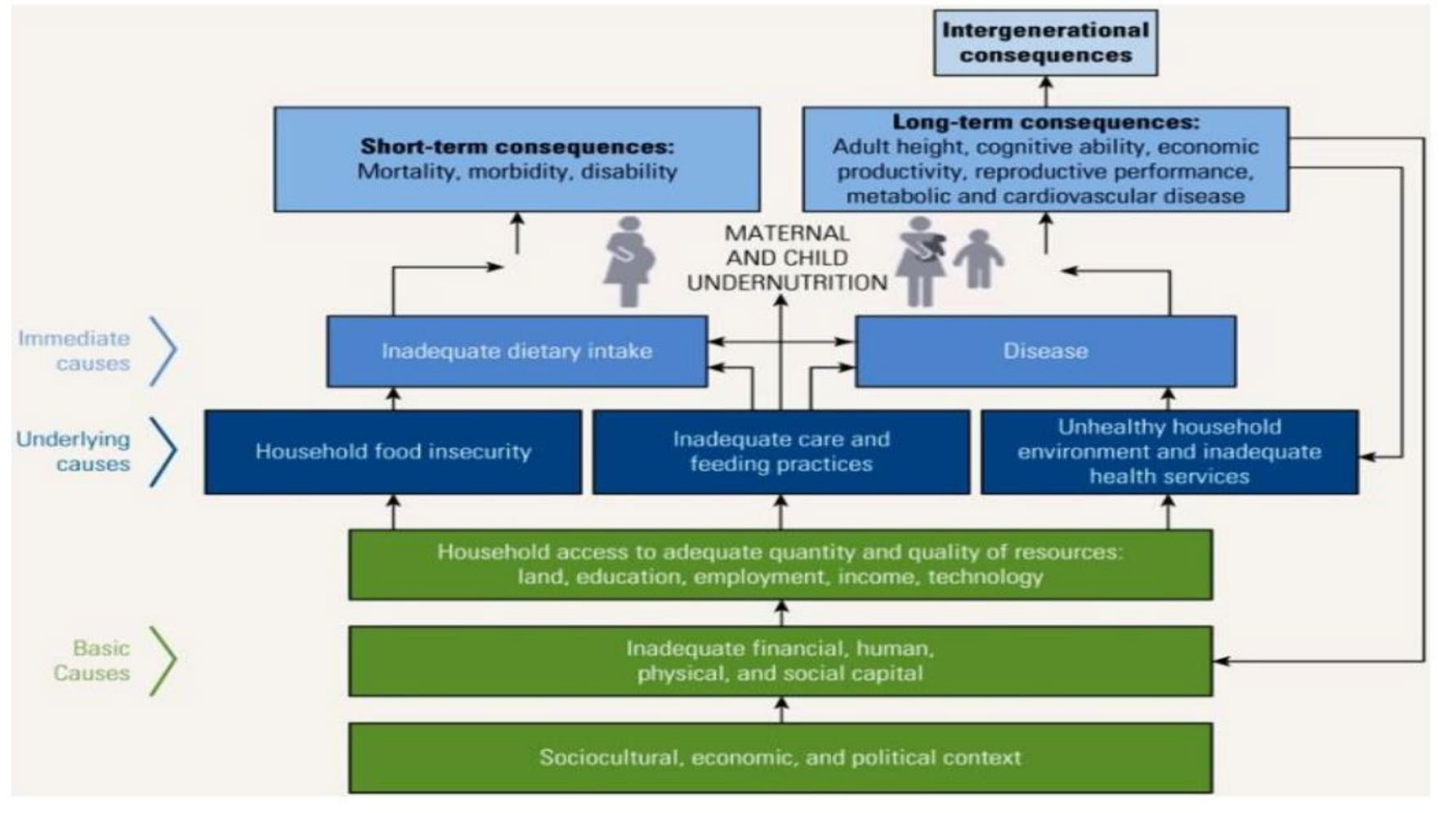
Conceptual framework of determinants of undernutrition (UNICEF 2013).

The objective of this study was to assess the determinants of severe acute malnutrition among children aged 6–36 months in riverine district of Ethiopia. This made necessary to investigate the determinants of severe acute malnutrition to uncover contextual factors based on UNICEF conceptual framework of determinants of undernutrition (see Fig. 1).

### Methods

All methods were carried out in accordance with relevant guidelines and regulations**.**

#### Study design and setting

This health facility based (health post) un-matched case control with qualitative study was conducted between December 2019 and January 2020 in Kalafo Woreda/district. Kalafo district is located and bounded between 655,013 and 541,626 m north, water is the river. The district has been designated as a malaria-prone area, and only 13.4% of households send their children to formal education, while 53% and 30.6% send their children to religious and in-formal school, respectively. Nearly half of Kelafo's population (46.3%) is food insecure, with 54% of households relying on food they produce and the rest relying on external sources^45^. According to an unpublished SMART survey report from 2018, the minimum dietary diversity and frequency were 26%, while exclusive breastfeeding and prevalence of households with hand washing facility was 51% and 7%.

From 32 Health posts (HPs) in Kalafo district, 27 functional HPs with OTP service were selected. Among the 27 functional HPs, only five HPs (Kalaman, Dariko, Helobacad, Bargun, and Allow-Igadsii) were randomly selected based on their catchment of under-five populations.

#### Eligibility criteria

**Figure 2:**
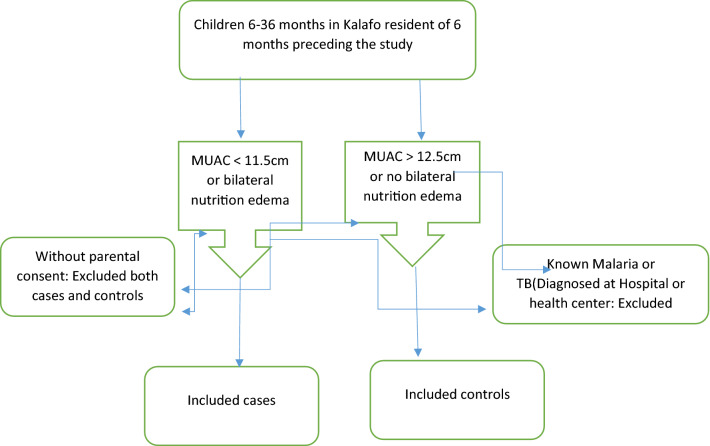
Eligibility flow chart for inclusion and exclusion of the study.

Children between 6 and 36 months in selected five health posts (HPs) of Kalafo district and lived the area past 6 months preceding the survey were study population. The cases were children between 6 and 36 months with mid-upper arm circumference (MUAC) < 11.5 cm or presence of bilateral nutritional edema. Controls were children between 6 and 36 months with MUAC ≥ 12.5 cm with no bilateral edema. Children with known diseases of tuberculosis and malaria and children without parental consent were excluded from the study (Fig. 2).

#### Sample size

The number of cases and controls required for the study were determined using Epi-Info version 3.5.4 StatCalc with 95% CL, 80% power, 1:2 case to control ratio and expected frequency of exposure in control group of 33.43%, to be able to detect a 2.24 odds ratio. The 33.43% exposure in control group was based on use of toilet facility as a determinant of infant nutritional status conducted in Dabat district, North Gondar, Ethiopia^29^ A total 246 children aged 6–36 months (82 cases, and 164 controls; 1:2 ratio) were studied.

#### Sampling procedure and data collection methods

The cases and controls were selected in 5 HPs of Kalafo district. The calculated sample size of cases (82) was allocated proportionally to each HP based on their estimated caseload; Kalaman (633.6), Dariko (753.4), Helobacad (631.7), Bargun (596) and Allow-Igadsii (813.5). Thus 15:30, 18:36, 15:30, 14:28 and 20:40 of eligible cases and controls were randomly selected from the five HPs respectively. Trained health extension workers and nurses who were working in each selected health posts were collected data under strict supervision.

The outcome of this study was severe acute malnutrition while socio-demographic status, demographic data, household structure, household hygiene/sanitation and maternal and child characteristics like feeding practices, presence of disease, health service utilization and household food security were as an independent variable.

Data on risk factor survey was conducted using standardized questionnaire (translated to Somali and back translation in to English) adapted from Conceptual framework of determinants of undernutrition (UNICEF, 2013) and other local studies on determinants of acute malnutrition^19,32,38^. 5% of questionnaire was pre-tested in Adis-Ketema HP of Kalafo district and approach and time per questionnaire were adjusted to improve data quality.

To assess dietary diversity practice, the sum of the number of different food groups consumed by the child in the 24 h prior to the survey were asked the mother or caretaker. The list of food groups were, grains, roots, and tubers; legumes and nuts; dairy products (milk, yogurt, cheese); flesh foods (meat, fish, poultry and liver/organ meats); eggs; vitamin-A rich fruits and vegetables and other fruits and vegetables. The respondents whose children consumed four or more of these food groups were labelled as having adequate dietary diversity and inadequate otherwise^35^.

The FANTA (Food and Nutrition Technical Assistance Tool) household food insecurity access scale (HFIAS) was used to assess food insecurity. It comprised of nine “occurrence questions” that represented a usually rising level of severity of food insecurity (access) and nine “frequency-of-occurrence” questions that inquired how often the condition happened as a follow-up to each occurrence question. If respondents stated that they had not experienced the condition mentioned in the relevant occurrence question in the preceding 4 weeks, the frequency-of-occurrence question was skipped (30 days). Finally, people were considered food secure if they responded “no” to all of the questions or only experienced worry on rare occasions and food insecure otherwise^35^. for further analysis, a food security index was created.

For qualitative part, KIIs were conducted using checklist extracted from UNICEF conceptual framework by experienced health workers familiar with local language and context. Local (Somali) language and tape record was used in all interviews. Key informant interview (KII) were conducted purposively on different individuals. The KIIs were HEWs, district nutrition focal person and nutrition working partners. The sample size of KII was determined by data saturation.

The child's nutritional condition was measured using MUAC tape placed halfway between the olecranon and acromion processes. According to the standard, the tape was comfortably positioned at the midpoint and verified to ensure that it was not pulled too tight or too loose while being recorded to the nearest 0.1 cm. Thumbs were lightly placed on the feet of each selected child bilaterally for three seconds to assess edema (count of 101, 102, and 103). Records were created as “normal”, “+”, “++”, and “+++” if no pitting, pitting on feet, feet plus legs, and feet plus hands and face, respectively, according to the standard when released pressing.

#### Operational definition

Diarrhea was determined when the child had three or more loose or watery stool in a 24-h’s period within the 2 weeks prior to the survey and this was assessed by asking the mothers or care takers^30^. Adequate dietary diversity is when children received at least four food groups out of seven in the preceding 24 h of the interview^35^. Food security defines the household food security level of the summations were ≤ 1 point out of 27 scores while the household food security level of the summations ≥ 2 points out of 27 scores were food insecure^36^.

#### Data management and analysis

Kobo toolbox was used for data entry and collection. Data was cleaned using Kobo toolbox and analyzed in SPSS version 20. Bivariate and multivariable binary logistic regression was employed to identify predictors. Of severe acute malnutrition. Variables with *P*-value < 0.25 in bivariate analysis and other important determinant of acute malnutrition were considered to Multivariate analysis. Whilst; variables with *P*-value < 0.05 were declared significant association with outcome variable in multivariate binary logistic regression analysis. Lastly, Strength of association was determined using the odds ratio with 95% CI, backward logistic regression method and Hosmer Lemeshow test for model goodness of fit. For qualitative data, it was transcribed manually and triangulated for better understanding about severe acute determinants.

### Ethical consideration

Ethical approval was obtained from research ethical committee of Jigjiga University, Ethiopia and the data was collected when written informed consent was obtained from each participant. The study was ethically cleared by college of medicine and health science methods section to this effect. Since data was collected from children signed informed consent of all parents was obtained. All authors consent to the publication of this manuscript.

## Results

### Socio-demographic characteristics of study participants in Kalafo district, Ethiopia

This study included 246 children aged 6–36 months of which 82 and 164 were cases and controls respectively. Among children, two of them had bilateral edema on feet whilst the rest had no edema. The mean age and standard deviation of cases and controls were 15.23 ± 7 and 19.6 ± 8.96 respectively. A small number of (19.5%) cases were older than 23 months compared to controls (40.2%). Proportion of children experienced diarrhea 71 (86.6) during the past 2 weeks and low birth interval 65 (79.3) was higher among cases compared controls. Whereas coverage of fully vaccinated 46 (56.1) and exclusively breastfed 36 (43.9) children were lower among cases compared to controls 110 (67.1) and 83 (50.6) respectively.

Furthermore, mothers in the control group received better 57 (69.5) nutritional counseling during ANC than women in the cases group 130 (79.3). However, the literacy and occupation of mothers in cases and controls were nearly same.

Regarding household characteristics, less children 50 (61%) among cases had adequate dietary diversity practices compared to controls 141 (86.0) and proportions of households with food insecurity were slightly higher among cases 29 (35.4%) than controls 63 (38.4%). Similarly, cases had a greater 52 (63.4%) percentage of three or more under 5-year-old children than controls 73 (44.5%). Only 57 (69.5%) of cases used treated water, compared to 117 (79.3%) of controls, and the use of latrines was comparable across cases and controls (Table 1).

**Table 1:** Socio-demographic characteristics of study participants in Kalafo district, Ethiopia.

Characteristics	Nutrition status	
**Child**		
Age	Case (%), n = 82	Control (%), n = 164
Mean age (± standard deviation)	15.23 (± 7)	19.6 (± 8.96)
Children aged 6–24 months	66 (81.4)	98 (59.8)
Sex of the child (Male)	42 (51.2)	89 (54.3)
Birth interval (< 2 years)	65 (79.3)	119 (72.6)
First child	11 (13.4)	24 (14.6)
Experienced diarrhea during the past 2 weeks	71 (86.6)	120 (73.2)
Fully vaccinated children	46 (56.1)	110 (67.1)
Exclusively breastfed during the first 6 months in child’s life	36 (43.9)	83 (50.6)
Received vitamin A supplementation in last 6 months	65 (79.3)	111 (67.7)
**Mother**		
Currently pregnant	57 (69.5)	115 (70.1)
Primary school or low	17 (20.7)	41 (25)
House wife	37 (45.1)	75 (45.7)
Nutrition counseling during ANC	57 (69.5)	130 (79.3)
Nutrition counseling during PNC	47 (57.3)	87 (53.0)
complementary Food demonstration	47 (57.3)	99 (60.4)
**Household**		
Number of under-five children ≥ 3 children	52 (63.4)	73 (44.5)
Household food insecure	29 (35.4)	63 (38.4)
Wash hands before eating	64 (78.0)	133 (81.1)
Adequate dietary diversity	50 (61.0)	141 (86.0)
Treat water	57 (69.5)	130 (79.3)
Have latrine	56 (68.3)	117 (71.3)
Waste disposal	82 (100)	151 (92.1)

### Determinants of severe acute malnutrition among children aged 6 to 36 months in Kalafo district, Ethiopia

All variables with a *P*-value of < 0.25 on bivariate analysis were included in the multivariate logistic regression model. Furthermore, given to their prominence in the literature, exclusive breastfeeding and male sex variables were included in the final model analysis, regardless of their *P*-value. χ^2^ (8, n = 246) = 8.747, *P*-value = 0.364, was used to test the goodness of fit model.

However, only the following five characteristics, were shown to be significantly associated with SAM among 6–59 aged children in Kalafo district: Children who had diarrhea two weeks prior to the research had nearly three times the chance of having severe acute malnutrition (cases). AOR = 2.85 (1.26, 6.45); 4.7 times among children eaten inadequate dietary diversity AOR = 4.68 (2.29, 9.58); 2.4 times higher among children whose mothers did not receive nutrition counseling during ANC visits AOR = 2.39 (1.16, 4.96); 2.3 times higher among children from households with more than three (3) under-five children, AOR = 2.28 (1.22, 4.26); and 3 times higher among children aged 6–11 and 12–17 months respectively, compared to controls (Table 2).

**Table 2:** Bivariate and multivariable analysis of factors associated with severe acute malnutrition among children aged 6 to 36 months in Kalafo district, Ethiopia.

Characteristics	Nutrition status		COR	AOR	*p*-value
**Age**	Case (%) n = 82	Control (%), n = 164			
6–11	28 (34.1)	37 (22.6)	3.12 (1.49, 6.51)	3.262 (1.46, 7.31)	0.004
12–17	29 (35.4)	33 (20.1 )	3.63 (1.73, 7.59)	3.237 (1.45, 7.23)	0.004
18–23	9 (11.0)	28 (17.1)	1.33 (0.52, 3.35)	0.79 (0.28, 2.21)	0.660
> 23	16 (19.5)	66 (40.2)	1	1	
**Experienced diarrhea during the past 2 weeks**					
No	11 (13.4)	44 (26.8)	1	1	
Yes	71 (86.6)	120 (73.2)	2.36 (1.15, 4.87)	2.85 (1.26, 6.45)	0.012
**Dietary diversity score**					
Adequate dietary diversity	50 (61.0)	141 (86.0)	1	1	
Inadequate dietary diversity	32 (39.0)	23 (14.0)	3.92 (2.09, 7.33)	4.68 (2.29, 9.58)	< 0.001
**Nutrition counseling during ANC**					
No	25 (30.5)	34 (20.7)	1.67 (0.92, 3.06)	2.39 (1.16, 4.96)	0.018
Yes	57 (69.5)	130 (79.3)	1		
**Number of under-five children**					
< 3 children	30 (36.6)	91 (55.5)	1		
≥ 3 children	52 (63.4)	73 (44.5)	2.16 (1.25, 3.73)	2.28 (1.22, 4.26)	0.010
**Sex of the child**					
Male	42 (51.2)	89 (54.3)	1	1	
Female	40 (48.8)	75 (45.7)	1.13 (0.66, 1.92)		
**Exclusively breastfed during the first 6 months in life**					
No	46 (56.1)	81 (49.4)	1.309 (0.77, 2.23)		
Yes	36 (43.9)	83 (50.6)	1	1	
**Birth order**					
First	11 (13.4)	24 (14.6)	1.19 (0.53, 2.68)		
Second and fifth	37 (45.1)	52 (31.7)	1.84 (1.03, 3.28)		
Above fifth	34 (41.5)	88 (53.7)	1	1	
**Do you treat your water**					
No	25 (30.5)	34 (20.7)	1.67 (0.92, 3.06)		
Yes	57 (69.5)	130 (79.3)	1	1	
**Fully vaccinated child**					
No	36 (43.9)	54 (32.9)	1.59 (0.92, 2.75)		
Yes	46 (56.1)	110 (67.1)	1	1	

### Determinants of severe acute malnutrition among children aged 6–36 months theme qualitative findings

In total, five KIIs were conducted; two HEWs KIIs, 2 active nutrition-working partners KIIs and one District nutrition focal person KII to deeply understand better the determinants of severe acute malnutrition in riverine context.

Qualitative study result showed a consistent finding with some identified significant determinants but not all. In general, qualitative study identified the following factors as a SAM determinant: contaminated water, Diarrhea, Polygamy, too number of children, farm chore and poor riverine communities.

“To my experience, SAM is common across children around the river (Negro) simply because they come across frequent diarrhea that resulted from drinking unclean river water. At the same time, most of the mothers are farm daily laborers and they may not have a time to take care their children. Therefore, I do give RUTF again to children who were successfully recovered from severe acute malnutrition previously”. HEW KII, Dariko HP.

In addition to this, “mothers in this area experience many children exceptionally and no mother takes family planning.” HEW KII, Kalaman HP.

“The people around this river do marry more than one wife and have many children from each wife and yet they do not have income to support, as they are farm daily laborers. Shebele river inhabitants (Negro) are not the farm owners but are daily workers.” Kalafo district Nutrition focal person KII.

“Previously there were many non-governmental organizations supporting the emergency nutrition sector. However; currently we are only one supporting and it is out of our capacity as severe acute malnutrition among children is continuously high across a year.” The cause of SAM in this area are many and main ones are diarrhea and lack of care. OTP Nurse, Save the children KII.

## Discussion

This quantitative study found that five factors were determining the severe acute malnutrition among children aged 6–36 months in riverine context. These were age group (6–11 and 12–17 months), inadequate dietary practices, and experienced diarrhea within two weeks preceding the data collection, from household with three and more under-five children and not exposed to nutrition counseling during ANC visits. However, poor childcare and polygamy are determinants of SAM in qualitative findings.

The odds of being severe acute malnutrition were higher among younger children aged 6–11 and 12–17 months compared to older children (18–23 and > 23 months). This finding is in line with studies done in Ethiopia^30^, Niger^39^, and Ghana^28^.

The possible explanation is the fact that most children do not go smoothly the transition period (initiation of complementary feeding) in low-income countries due to many reasons.

The odds of being severe acute malnutrition were higher among children from households with three or more under-five children compared with children from households with less than three under-five children. This finding is consistent with studies done in India^21^, Ethiopia^14,33,40^, and report of Ethiopian DHS 2016^41^. The finding of our study is also in agreement with studies done in Ethiopia^30–32^ which indicates larger family size (≥ 4 or 5) was determining factor for wasting among under-five children. The possible explanation is that short birth interval is positively correlated with malnutrition among under five children, which means children with short birth interval has higher risk of malnutrition^10,46,47^. It may also due to households either with higher number of under- five children or larger family size increase chore on mothers, which compromise the care or feeding practices of their children^31^.

However, this finding is against a study done in Arizona, USA^23^. This might be due to a difference in context, study design and outcome (SAM) of current study.

The current study shows that odds of being severe acute malnutrition were higher among children who had inadequate dietary diversity in past 24 h preceding the data collection compared their counterparts. This finding is in line with studies done in India^21^, Afghanistan^26^ and Ethiopia^9,31^.

However, it contradicts studies done in Cambodia^42^ and Tanzania^43^ where inadequate dietary diversity was not associated with acute malnutrition of under-five children. The only possible explanation could be contextual factors that might determine acute malnutrition.

The current study also shows that odds of being severe acute malnutrition were higher among children who experienced diarrhea in past two weeks preceding the data collection compared their counterparts. This finding is in line with studies done in Afghanistan^26^, Ethiopia^17,27,32^ and Niger^38^. The possible explanation is environmental enteric dysfunction (EED) as children in the study area expose inadequate water, sanitation and hygiene. EED is a complex digestive condition caused by long-term exposure to enteric infections. It may also due to low coverage of rotavirus vaccine among children in Kalafo. Recent studies in low and middle income countries show that SAM and EED among children has vicious cycle^48,49^.

Lastly, the odds of being severe acute malnutrition were higher among children whose mothers did not expose to nutrition counseling during ANC visits. This study is in line with studies done in Ethiopia^29,33^. However, study done in Pakistan^44^ shows counseling during ANC associated with stunting among under five children but not wasting. This might be due to different livelihood (riverine) of the current study and the fact that underlying factors (infant and young child feeding counseling) of undernutrition might not directly results acute malnutrition.

The finding of qualitative study shows poor childcare and polygamy were the determinants of SAM. However; according to quantitative study, the main determinants of severe acute malnutrition in riverine context were diarrhea and inadequate dietary intake under the category of immediate cause of malnutrition. Whilst higher (≥ 3) number of under-five children were another important contextual determinant of severe acute malnutrition among children (6–36 months) in riverine zone of Somali region. Briefly, young age (6–11 and 12–17 months) and not having nutrition counseling during pregnancy were also among distal determinants of severe acute malnutrition in riverine context.

Improvement has to be made on childcare giving practices including dietary diversity. Water treatment and family planning intervention should be integrated along with community-based management of severe acute malnutrition (CMAM) programs in riverine context.

The researchers employed both quantitative and qualitative methods to discover the factors that influence SAM in children under the age of five in a river setting. This can be used as an input in nutrition initiatives by programmers and policymakers. However, there are some drawbacks to the study: determinant variables including diarrhea, dietary habit, and food security were examined by questioning respondents, which could expose social desirability and recall bias. The data was collected by trained and experienced health professional in the HPs to manage the study's potential weakness.

## Data Availability

The data is available upon reasonable request at correspondence author.

## Abbreviations

CMAM: Community Based Management of Acute Malnutrition
ANC: Antenatal care
HP: Health post
EDHS: Ethiopian Demographic and Health Survey
IFPRI: International food and policy research institute
DDS: Dietary diversity score
FANTA: Food and Nutrition Technical Assistance
HFIAS: Household food insecurity access scale
MUAC: Mid upper arm circumference
SAM: Severe acute malnutrition

## References

[1] Akter, S., Roy, S., Thakur, S., Sultana, M., Khatun, W., Rahman, R., *et al*. *Does Market Access Mitigate the Impact of Seasonality on Child Growth? Panel Data Evidence from Northern Ethiopia*. Innocenti Working Paper WP-2016–05. Florence, Italy: UNICEF Office of Research Policy **35**(4), 286–93 (2016)

[2] Hemalatha R, Pandey A, Kinyoki D, Ramji S, Lodha R, Kumar GA (2020). Mapping of variations in child stunting, wasting and underweight within the states of India: the Global Burden of Disease Study 2000–2017. EClinicalMedicine.

[3] von Grebmer, K., Saltzman, A., Birol, E., Wiesman, D., Prasai, N., Yin, S., *et al*. *Global Hunger Index: The Challenge of Hidden Hunger*. IFPRI books (2014)

[4] Bhandari TR, Chhetri M (2013). Nutritional status of under five year children and factors associated in Kapilvastu District, Nepal. J Nutr Health Food Sci..

[5] World Health Organization. *Pocket book of hospital care for children: guidelines for the management of common childhood illnesses*. World Health Organization (2013). 24006557

[6] Estimation UI-aGfCM. L*evels & Trends in Child Mortality: Report 2012*. IGME (2012).

[7] Hoffman D, Cacciola T, Barrios P, Simon J (2017). Temporal changes and determinants of childhood nutritional status in Kenya and Zambia. J. Health Popul. Nutr..

[8] Teshale AB, Tesema GA (2020). Prevalence and associated factors of delayed first antenatal care booking among reproductive age women in Ethiopia; a multilevel analysis of EDHS 2016 data. PloS one..

[9] Tariku A, Bikis GA, Woldie H, Wassie MM, Worku AG (2017). Child wasting is a severe public health problem in the predominantly rural population of Ethiopia: A community based cross–sectional study. Arch. Public Health.

[10] Egata G, Berhane Y, Worku A (2014). Predictors of acute undernutrition among children aged 6 to 36 months in east rural Ethiopia: a community based nested case-control study. BMC Pediatr..

[11] Eshete H, Abebe Y, Loha E, Gebru T, Tesheme T (2017). Nutritional status and effect of maternal employment among children aged 6–59 months in Wolayta Sodo Town, Southern Ethiopia: A cross-sectional study. Ethiop. J. Health Sci..

[12] Farah, A. E, Abas, A. H. & Ahmed, A. T. *Bottlenecks and met needs for the treatment of severe acute malnutrition in pastoralists: Doolo zone of Somali region, Ethiopia* (2020)

[13] Gelu A, Edris M, Derso T, Abebe Z (2018). Undernutrition and associated factors among children aged 6–59 months living in slum areas of Gondar city, northwest Ethiopia: a cross-sectional study. Pediatr. Health Med. Therap..

[14] Fentaw R, Bogale A, Abebaw D (2013). Prevalence of child malnutrition in agro-pastoral households in Afar Regional State of Ethiopia. Nurs. Res. Pract..

[15] Pravana NK, Piryani S, Chaurasiya SP, Kawan R, Thapa RK, Shrestha S (2017). Determinants of severe acute malnutrition among children under 5 years of age in Nepal: a community-based case–control study. BMJ Open.

[16] van Cooten MH, Bilal SM, Gebremedhin S, Spigt M (2019). The association between acute malnutrition and water, sanitation, and hygiene among children aged 6–59 months in rural Ethiopia. Maternal Child Nutr..

[17] Gone T, Lemango F, Eliso E, Yohannes S, Yohannes T (2017). The association between malaria and malnutrition among under-five children in Shashogo District, Southern Ethiopia: a case-control study. Infect. Dis. Poverty.

[18] Seid A, Seyoum B, Mesfin F (2017). Determinants of acute malnutrition among children aged 6–59 months in Public Health Facilities of Pastoralist Community, Afar Region, Northeast Ethiopia: A case control study. J. Nutr. Metabol..

[19] Burza S, Mahajan R, Marino E, Sunyoto T, Shandilya C, Tabrez M (2016). Seasonal effect and long-term nutritional status following exit from a community-based management of severe acute malnutrition program in Bihar, India. Eur. J. Clin. Nutr..

[20] Li P-J, Jin T, Luo D-H, Shen T, Mai D-M, Hu W-H (2015). Effect of prolonged radiotherapy treatment time on survival outcomes after intensity-modulated radiation therapy in nasopharyngeal carcinoma. PloS one..

[21] Ambadekar N, Zodpey S (2017). Risk factors for severe acute malnutrition in under-five children: A case–control study in a rural part of India. Public Health.

[22] Awoke A, Ayana M, Gualu T (2018). Determinants of severe acute malnutrition among under five children in rural Enebsie Sarmidr District, East Gojjam Zone, North West Ethiopia, 2016. BMC Nutr..

[23] Costa ME, Trumble B, Kaplan H, Gurven MD (2018). Child nutritional status among births exceeding ideal family size in a high fertility population. Maternal Child Nutr..

[24] Abebe Z, Haki GD, Baye K (2018). Simulated effects of home fortification of complementary foods with micronutrient powders on risk of inadequate and excessive intakes in West Gojjam, Ethiopia. Maternal Child Nutr..

[25] Deribew A, Alemseged F, Tessema F, Sena L, Birhanu Z, Zeynudin A (2010). Malaria and under-nutrition: A community based study among under-five children at risk of malaria, south-west Ethiopia. PLos one..

[26] Frozanfar MK, Yoshida Y, Yamamoto E, Reyer JA, Dalil S, Rahimzad AD (2016). Acute malnutrition among under-five children in Faryab, Afghanistan: Prevalence and causes. Nagoya J. Med. Sci..

[27] Motbainor A, Taye A (2019). Wasting in under five children is significantly varied between rice producing and non-producing households of Libokemkem district, Amhara region, Ethiopia. BMC Pediatr..

[28] Boah M, Azupogo F, Amporfro DA, Abada LA (2019). The epidemiology of undernutrition and its determinants in children under five years in Ghana. Plos One.

[29] Wubante AA (2017). Determinants of infant nutritional status in Dabat district, North Gondar, Ethiopia: A case–control study. PloS One..

[30] Karthik L, Kumar G, Keswani T, Bhattacharyya A, Chandar SS, Rao KB (2014). Protease inhibitors from marine actinobacteria as a potential source for antimalarial compound. PloS one..

[31] Abate KH, Belachew T (2017). Care and not wealth is a predictor of wasting and stunting of ‘The Coffee Kids’ of Jimma Zone, southwest Ethiopia. Nutr. Health.

[32] Gebre A, Reddy PS, Mulugeta A, Sedik Y, Kahssay M (2019). Prevalence of malnutrition and associated factors among under-five children in pastoral communities of Afar Regional State, Northeast Ethiopia: A community-based cross-sectional study. J. Nutr. Metabol..

[33] Wie GT, Tsegaye D (2020). Determinants of acute malnutrition among children aged 6–59 months visiting public health facilities in Gambella Town, Southwest Ethiopia: Unmatched case–control study. Nutr. Diet. Suppl..

[34] Ahmed, A.T., Elmi, A., Abas, A. & Omer, A. *Determinants of severe acute malnutrition among children 6–36 months in Kalafo District, Riverine Context, Ethiopia*. Unmatched Case Control With Qualitative Study (2020).

[35] Worku T, Gonete KA, Muhammad EA, Atnafu A (2020). Sustainable under nutrition reduction program and dietary diversity among children’s aged 6–23 months, Northwest Ethiopia: Comparative cross-sectional study. Int. J. Equity Health.

[36] Coates, J., Swindale, A. & Bilinsky, P. *Household Food Insecurity Access Scale (HFIAS) for measurement of food access: Indicator guide: Version 3* (2007).

[37] Fikrie A, Alemayehu A, Gebremedhin S (2019). Treatment outcomes and factors affecting time-to-recovery from severe acute malnutrition in 6–59 months old children admitted to a stabilization center in Southern Ethiopia: A retrospective cohort study. Ital. J. Pediatr..

[38] Fava, F. P., Upton, J., Banerjee, R. R., Taye, M., & Mude A. G. *Pre-feasibility study for index-based livestock insurance in Niger* (2018).

[39] Fava, F., Upton, J., Banerjee, R., Taye, M. & Mude, A. *Pre-feasibility study for Index-Based Livestock Insurance in Niger*. ILRI Research (2018).

[40] Demissie S, Worku A (2013). Magnitude and factors associated with malnutrition in children 6–59 months of age in pastoral community of Dollo Ado district, Somali region, Ethiopia. Sci. J. Public Health.

[41] Edhs E (2016). demographic and health survey 2016: Key indicators report. DHS Progr. ICF.

[42] McDonald C, McLean J, Kroeun H, Talukder A, Lynd L, Green T (2015). Household food insecurity and dietary diversity as correlates of maternal and child undernutrition in rural Cambodia. Eur. J. Clin. Nutr..

[43] Khamis AG, Mwanri AW, Ntwenya JE, Kreppel K (2019). The influence of dietary diversity on the nutritional status of children between 6 and 23 months of age in Tanzania. BMC Pediatr..

[44] Khan S, Zaheer S, Safdar NF (2019). Determinants of stunting, underweight and wasting among children < 5 years of age: Evidence from 2012–2013 Pakistan demographic and health survey. BMC Public Health.

[45] Dube KD, Reddy UR, Girmay A (2017). Tribes perception for agriculture and rural house hold food in-security in Kelafo Woreda. Gode Zone Somali Reg..

[46] Sultana P, Rahman M, Akter J (2019). Correlates of stunting among under-five children in Bangladesh: A multilevel approach. BMC Nutr..

[47] Rahman MS, Howlader T, Masud MS, Rahman ML (2016). Association of low-birth weight with malnutrition in children under five years in Bangladesh: do mother’s education, socio-economic status, and birth interval matter?. PloS One.

[48] Koyuncu A, Simuyandi M, Bosomprah S, Chilengi R (2020). Nutritional status, environmental enteric dysfunction, and prevalence of rotavirus diarrhoea among children in Zambia. PloS One.

[49] Prendergast AJ, Kelly P (2016). Interactions between intestinal pathogens, enteropathy and malnutrition in developing countries. Curr. Opin. Infect. Dis..

